# Impact of non-regional lymph node metastases accurately revealed on ^18^F-PSMA-1007 PET/CT in the clinical management of metastatic hormone-sensitive prostate cancer

**DOI:** 10.1186/s13550-023-01009-x

**Published:** 2023-07-06

**Authors:** Zhangdong Jiang, Junjie Fan, Chaosheng Gan, Xiaoxin Dong, Guoqiang Gao, Zhuonan Wang, Dalin He, Lei Li, XiaoYi Duan, Kaijie Wu

**Affiliations:** 1 Department of Urology, The First Affiliated Hospital of Xi’an Jiaotong University, #277 Yanta West Road, Xi’an, 710061 Shaanxi People’s Republic of China; 2 Department of Urology, Baoji Central Hospital, Baoji, Shaanxi People’s Republic of China; 3 Department of PET/CT Imaging, The First Affiliated Hospital of Xi’an Jiaotong University, #277 Yanta West Road, Xi’an, 710061 Shaanxi People’s Republic of China

**Keywords:** ^18^F-PSMA-1007 PET/CT, Conventional imaging, Non-regional lymph node metastases, Docetaxel, mHSPC

## Abstract

**Background:**

Non-regional lymph node (NRLN) metastases has shown increasing importance in the prognosis evaluation and clinical management of primary metastatic hormone-sensitive prostate cancer (mHSPC). Hence, this study aimed to investigate the concordance rates between ^18^F-PSMA-1007 PET/CT and conventional imaging (CI) in revealing NRLN metastases, and explore the impact of NRLN metastases on the management of primary mHSPC.

**Methods:**

The medical records of 224 patients with primary mHSPC were retrospectively reviewed, including 101 patients (45.1%) only received CI for TNM classification, 24 patients (10.7%) only received ^18^F-PSMA-1007 PET/CT, and 99 patients (44.2%) received both ^18^F-PSMA-1007 PET/CT and CI. Among patients who received ^18^F-PSMA-1007 PET/CT and CI before initial treatment, the concordance rates between ^18^F-PSMA-1007 PET/CT and CI were analyzed. The high-volume disease was defined as the presence of visceral metastases and/or ≥ 4 bone metastases (≥ 1 beyond the vertebral bodies or the pelvis) based on the findings of ^18^F-PSMA-1007 PET/CT and/or CI. The primary endpoint was progression-free survival (PFS), and Cox regression analyses were performed to explore independent predictors of PFS.

**Results:**

A total of 99 patients (44.2%) received both ^18^F-PSMA-1007 PET/CT and CI, the concordance rate in revealing NRLN metastases between ^18^F-PSMA-1007 PET/CT and CI was only 61.62%, and Cohen’s kappa coefficient was as low as 0.092. Moreover, ^18^F-PSMA-1007 PET/CT detected an additional 37 of 94 (39.4%) patients with positive NRLNs who were negative on CI. Cox regression revealed that androgen deprivation therapy (ADT), N1, high-volume, NRLN and visceral metastases were associated with worse PFS (all *P* < 0.05) in 224 patients. Furthermore, in patients with low-volume disease, the median PFS of patients with NRLN metastases was significantly shorter than that of patients without NRLN metastases (19.5 vs. 27.5 months, *P* = 0.01), while the difference between patients with low-volume plus NRLN metastases and high-volume disease was not significant (19.5 vs. 16.9 months, *P* = 0.55). Moreover, early docetaxel chemotherapy significantly prolonged the PFS of these patients compared with ADT alone (20.7 vs. 12.3 months, *P* = 0.008).

**Conclusion:**

NRLN metastases could be accurately revealed by ^18^F-PSMA-1007 PET/CT, which should be considered a high-volume feature, especially concomitant with bone metastases. Furthermore, patients with low-volume plus NRLN metastases may be suitable for more intensive treatment, such as early docetaxel chemotherapy.

**Supplementary Information:**

The online version contains supplementary material available at 10.1186/s13550-023-01009-x.

## Background

Prostate cancer (PCa) is the second most common cancer in men worldwide, and approximately 68% of primary patients present with metastatic hormone-sensitive PCa (mHSPC) in China [1, 2]. Androgen deprivation therapy (ADT) is the mainstay therapy for primary mHSPC, while the durability of response to ADT and time to castration resistance is variable owing to the significant heterogeneity of mHSPC [3–5]. In the past few years, the treatment paradigm of mHSPC has tremendously changed, and docetaxel, abiraterone and enzalutamide have been sequentially approved as first-line treatment options for primary mHSPC [6].

As proposed in the CHAARTED trials, the localization and number of metastases are widely used to categorize mHSPC into low- or high-volume disease for risk stratification and treatment selection purposes [7]. As we all know, the common sites of metastasis from PCa, in order of decreasing frequency, are the bone (84%), distant lymph nodes (10.6%), liver (10.2%) and thorax (9.1%) [8]. However, this classification only focused on the status of bone and visceral metastases and did not include non-regional lymph node (NRLN) metastases. Emerging evidence suggests that NRLN metastases is an important prognostic factor for mHSPC, especially concomitant with bone metastases [9, 10]. Furthermore, a study conducted by Shiota et al. showed that NRLN metastases were associated with worse progression-free survival (PFS) than bone metastases, especially in patients with low-volume diseases [11]. Thus, in addition to bone or visceral metastases, NRLN metastases should be included as another risk factor to classify low- or high-volume disease, but the clinical application value of NRLN metastases is limited by its detection rate, which is as low as 9–12% [12, 13].

Currently, conventional imaging (CI) modalities, including computerized tomography (CT) or magnetic resonance imaging (MRI), are recommended to evaluate metastatic spread to lymph nodes, and morphological changes in lymph nodes are the main diagnostic criterion [14, 15]. However, morphologic changes frequently lag behind functional changes, making it difficult for CI to identify small metastatic lesions and distinguish metastases from inflammation, which leads to the low sensitivity and specificity of CI in detecting NRLN metastases [16]. Prostate-specific membrane antigen (PSMA) is expressed on PCa cells and often shows marked overexpression in metastatic PCa tissue [17, 18]. Therefore, PSMA PET/CT may be a more promising technique for accurately detecting NRLN metastases than CI. Furthermore, the definition of low- and high-volume disease in the CHAARETED trial was based upon CI, and studies exploring the concordance rates between PSMA PET/CT and CI in terms of tumor volume evaluation are limited. To address this void, we analyzed a consecutive cohort of patients with primary mHSPC who received PSMA PET/CT or CI before initial treatment. We hypothesized that PSMA PET/CT could detect more NRLN metastases, which might affect tumor volume evaluation and treatment selection.

## Methods

### Patients and study design

From January 2019 to December 2021, all patients with primary mHSPC who underwent ^18^F-PSMA-1007 PET/CT or CI before initial treatment were identified in our database. Patients who had received local or systemic treatment before imaging examination, had prior invasive malignancy or any serious comorbidity and had incomplete data were excluded. A total of 224 patients were included in the final analyses. This study was approved by the Ethics Committee of the First Affiliated Hospital of Xi'an Jiaotong University, Xi'an, P.R. China. All subjects signed an informed consent form.

Basic information, laboratory values, and imaging examination findings, such as age, biopsy Gleason score (GS), prostate-specific antigen (PSA) at diagnosis, primary treatment and imaging examination modality, metastatic sites, etc., of these patients were retrospectively reviewed.

According to CHAARTED, the high-volume disease was defined as the presence of visceral metastases and/or ≥ 4 bone metastases (≥ 1 beyond the vertebral bodies or the pelvis) based on the findings of ^18^F-PSMA-1007 PET/CT and/or CI [7]. Patients who did not meet the criteria of high-volume disease were defined as low-volume disease. For patients who received both ^18^F-PSMA-1007 PET/CT and CI before initial treatment, the discrepancies between ^18^F-PSMA-1007 PET/CT and CI were resolved by discussion.

### Conventional imaging

CI included chest, abdominal, and pelvic cross-sectional imaging (i.e., CT or MRI) and Tc-99 m bone scintigraphy. When dimensions on lymph nodes were available, a lymph node with a short axis of ≥ 1 cm was considered suspicious for metastasis. Otherwise, a lymph node was deemed “positive” if classified as such by the interpreting radiologist in the clinical report.

### ^18^F-PSMA-1007 acquisition and imaging analysis

^18^F-PSMA-1007 was synthesized as described in the literature [19]. The reagent kits, PSMA-1007 precursor, and PSMA-1007 reference standard were purchased from ABX Advanced Biochemical Compounds GmbH, Germany. A whole-body PET/CT scan was conducted using the GEMINI TF64 PET/CT system (Philips Health care). ^18^F-PSMA-1007 was injected intravenously at a weight-based dose of 97–316 MBq for 90 min before acquisition began. The variation in injected dose was due to the large-scale patients serving at each production (18–20 patients), resulting in lower activity for the last one or two patients each time. However, the image quality of the PET scans in patients administered low activity was good and judged sufficient for accurate image analysis [20]. All ^18^F-PSMA-1007 PET/CT images were analyzed using Fusion Viewer software in the Extended Brilliance Workstation (EBW, Philips, Netherlands). Lesions in the whole body with uptake above the background activity were defined as metastases. All PET/CT images were reviewed by 2 blinded nuclear physicians, and discrepancies were resolved by discussion.

### Follow-up and outcome measures

Patients were followed up every month for the first 3 months and every 3 months thereafter with PSA tests and CI examinations. NRLN metastases included positive lymph nodes of the abdominal, inguinal, and upper diaphragm. For patients who received ^18^F-PSMA-1007 PET/CT and CI before initial treatment, the concordance rate of ^18^F-PSMA-1007 PET/CT with CI for the identification of T and N stage and metastatic sites was analyzed. The endpoint of survival evaluation was PFS, which was defined as the time from treatment beginning to biochemical and/or clinical/radiological progression (based on the results of CI examinations) or cancer-specific death, whichever occurred first [21].

### Statistical analysis

Data for continuous variables are presented as the mean ± standard deviation, and categorical data are displayed as numbers (percentage). The concordance rates between ^18^F-PSMA-1007 PET/CT and CI were assessed by Cohen’s kappa coefficient. Cox regression analyses were performed to explore independent predictors of PFS. Survival curves were generated using the Kaplan‒Meier method, and log-rank tests were used to compare the differences. Statistical analysis was performed using SPSS 23.0 for Windows (SPSS, Chicago, IL). All tests were 2-tailed, and *p* < 0.05 was considered to be statistically significant.

## Results

### Baseline characteristics

As shown in Table 1, the mean age and PSA at diagnosis of the 224 patients were 70.27 ± 8.85 and 531.02 ± 1055.28 ng/mL, respectively. A total of 142 patients (63.4%) had biopsy GSs of 9–10, and 132 patients (58.9%) had high-volume disease. Ninety-four patients (42.0%) were treated with ADT, and 130 patients (58.0%) were treated with ADT combined with docetaxel. Before treatment, 101 patients (45.1%) only received CI for TNM classification, 24 patients (10.7%) only received ^18^F-PSMA-1007 PET/CT, and 99 patients (44.2%) received both ^18^F-PSMA-1007 PET/CT and CI. Then, 162 patients (72.3%) were diagnosed with clinical tumor T stage ≥ 3, and 139 patients (62.1%) were positive for clinical N stage (N1). Moreover, NRLN metastases were found in 55 patients (24.6%), bone metastases were found in 208 patients (92.9%), and visceral metastases were found in 22 patients (9.8%). The median follow-up and PFS of 224 patients were 27.38 ± 8.69 and 17.56 ± 8.32 months, respectively.

**Table 1:** Baseline characteristics

Variable	Total (*N* = 224)
Age, years (mean ± SD)	70.27 ± 8.85
PSA at diagnosis, ng/mL (mean ± SD)	531.02 ± 1055.28
Biopsy Gleason score, n (%)	
6–7	26 (11.6)
8	56 (25.0)
9–10	142 (63.4)
Imaging modality, n (%)	
CI	101 (45.1)
^18^F-PSMA-1007 PET/CT	24 (10.7)
Both CI and ^18^F-PSMA-1007 PET/CT	99 (44.2)
Primary treatment modality, n (%)	
ADT	94 (41.9)
ADT + Docetaxel	130 (58.1)
Tumor volume, n (%)	
Low	92 (41.1)
High	132 (58.9)
T stage, n (%)	
cT2	62 (27.7)
cT3	80 (35.7)
cT4	82 (36.6)
N stage, n (%)	
N0	85 (37.9)
N1	139 (62.1)
Metastasis site, n (%)	
NRLN	55 (24.6)
Bone	208 (92.9)
Visceral	22 (9.8)
Median PFS, months (mean ± SD)	17.56 ± 8.32
Median follow-up, months (mean ± SD)	27.38 ± 8.69

*PSA* prostate specific antigen, *CI* conventional imaging, *PSMA* prostate specific membrane antigen, *ADT* androgen deprivation therapy, *NRLN* non-regional lymph node, *PFS* progression-free survival

### Concordance rate between CI and ^18^F-PSMA-1007 PET/CT

**Fig. 1:**
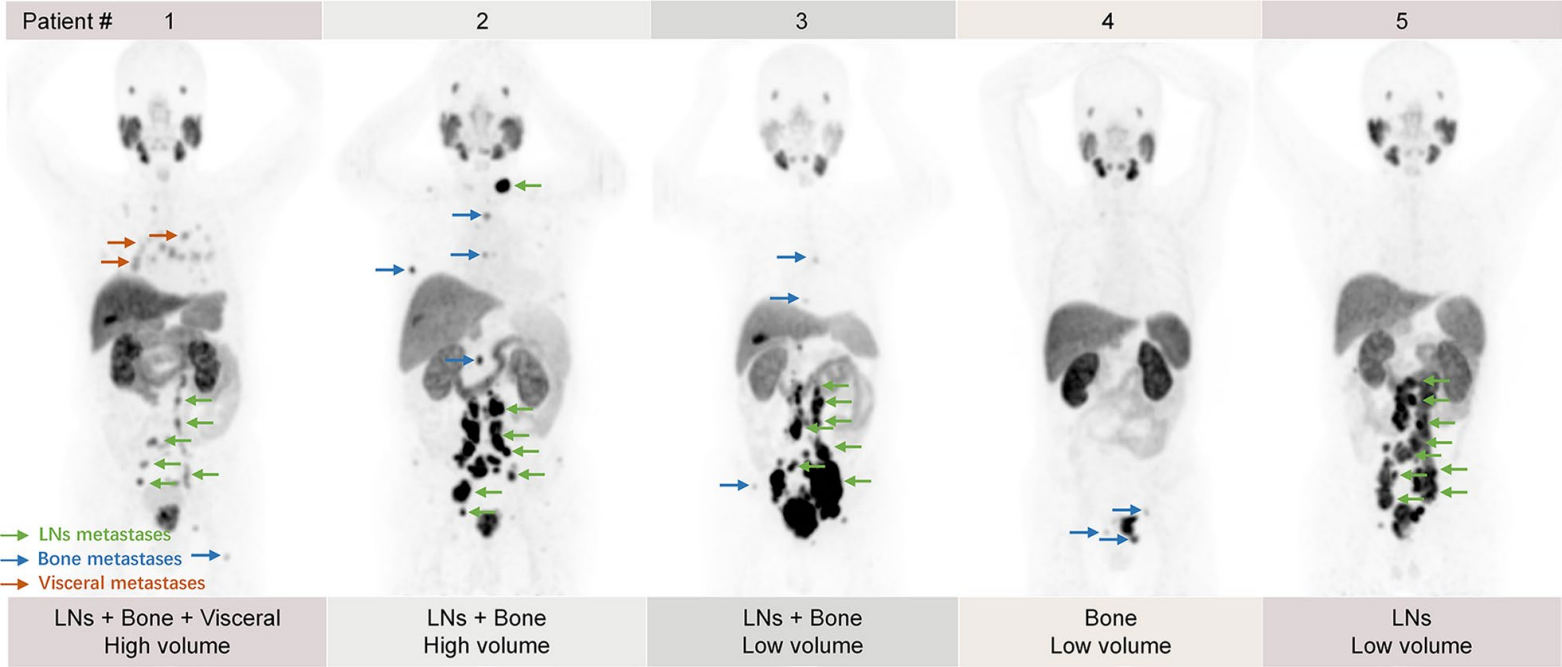
Representative images of metastasis distribution on ^18^F-PSMA-1007 PET/CT in mHSPC with different tumor volumes

Representative images of metastasis distribution on ^18^F-PSMA-1007 PET/CT in mHSPC with different tumor volumes are shown in Fig. 1. Among 99 patients who received both ^18^F-PSMA-1007 PET/CT and CI before treatment, there were 19 patients with miT2 (19.19%), 36 patients with miT3 (36.36%) and 44 patients with miT4 (44.45%), and there were 19 patients with miN0 (19.19%), 37 patients with miN1 (37.37%) and 43 patients with miN2 (43.44%). Furthermore, the concordance rates between CI and ^18^F-PSMA-1007 PET/CT in terms of tumor volume, T stage, N stage, bone metastases, and visceral metastases were 90.91%, 89.90%, 84.85%, 82.83%, and 86.87%, respectively (Table 2). However, the concordance rate in revealing NRLN metastases was only 61.62%, and Cohen’s kappa coefficient was as low as 0.092.

**Table 2:** The concordance rate between ^18^F-PSMA-1007 PET/CT and CI

	CI	^18^F-PSMA-1007 PET/CT			Concordance rate (%)	Cohen's kappa coefficient
Tumor volume		Low	High		90.91	0.816
	Low	38	9			
	High	0	52			
T stage		miT2	miT3	miT4	89.90	0.844
	cT2	19	6	1		
	cT3	0	30	3		
	cT4	0	0	40		
N stage		miN0	miN1	miN2	84.85	0.600
	cN0	17	9	4		
	cN1	2	28	39		
NRLN metastases		Absence	Presence		61.62	0.092
	Absence	57	37			
	Presence	1	4			
Bone metastases		Absence	Presence		82.83	0.456
	Absence	10	16			
	Presence	1	70			
Visceral metastases		Absence	Presence		86.87	0.207
	Absence	84	13			
	Presence	0	2			

*CI* conventional imaging, *PSMA* prostate specific membrane antigen, *NRLN* non-regional lymph node

### The difference of NRLN metastases detection rates between CI and ^18^F-PSMA-1007 PET/CT

The detection rates of NLRN metastases for ^18^F-PSMA-1007 PET/CT and ^18^F-PSMA-1007 PET/CT + CI were 33.33% (8/24), 42.42% (42/99), respectively. However, the detection rates of NRLN metastases for CI was only 4.95% (5/101), which was significantly lower than ^18^F-PSMA-1007 PET/CT and ^18^F-PSMA-1007 PET/CT + CI (4.95% vs. 33.33%, 4.95% vs. 42.42%, *P* < 0.05). Among 123 patients who received ^18^F-PSMA-1007 PET/CT before treatment, the patient-based analysis showed that the detection rate of NRLN metastases was 39.83% (49/123). However, the detection rate of NRLN metastases was only 5.00% (10/200) in 200 patients who received CI before treatment, which was significantly lower than ^18^F-PSMA-1007 PET/CT (5.00% vs. 39.83%, *P* < 0.05). Furthermore, the lesion-based analysis also showed that the detection rates of NLRN metastases for CI was significantly lower than ^18^F-PSMA-1007 PET/CT (3.87% vs. 36.24%, *P* < 0.05).

### Prognostic factors of PFS in primary mHSPC

Of the 224 patients included in the present study, 146 patients (65.2%) had diseases progression during the period of follow-up. Univariate Cox regression revealed that primary treatment modality, tumor volume, N stage, and the metastatic status of NRLNs and visceral organs were associated with PFS (Table 3). After reintegrating all the above five variables in the Cox regression model, the multivariate analysis suggested that ADT, N1, high-volume and visceral metastases were also associated with worse PFS (all *P* < 0.05). Furthermore, the risk of disease progression was decreased by half in the absence of NRLN metastases.

**Table 3:** Cox regression analysis for associations of clinicopathological parameters with PFS

Variable	Univariable			Multivariable		
	HR	95%CI	*P*	HR	95%CI	*P*
Age (continuous)	1.001	0.982–1.021	0.886			
PSA at diagnosis (continuous)	1.021	0.864–1.473	0.378			
Biopsy Gleason score						
8 vs. 6–7	1.39	0.758–2.548	0.288			
9–10 vs. 6–7	1.366	0.793–2.350	0.261			
Primary treatment modality (ADT vs. ADT + Docetaxel)	0.534	0.384–0.742	0.001	0.318	0.219–0.461	0.001
Tumor volume (low vs. high)	0.376	0.263–0.537	0.001	0.383	0.264–0.556	0.001
T stage						
cT3 vs. cT2	1.392	0.916–2.115	0.121			
cT4 vs. cT2	1.244	0.813–1.903	0.315			
N stage (N0 vs. N1)	0.551	0.386–0.787	0.001	0.501	0.339–0.740	0.001
NRLN metastases (absence vs. presence)	0.522	0.356–0.764	0.001	0.407	0.265–0.625	0.001
Bone metastases (absence vs. presence)	0.833	0.470–1.477	0.532			
Visceral metastases (absence vs. presence)	0.38	0.227–0.635	0.001	0.346	0.196–0.613	0.001

*PSA* prostate specific antigen, *ADT* androgen deprivation therapy, *NRLN* non-regional lymph node, *PFS* progression-free survival

### Exploratory analysis

**Fig. 2:**
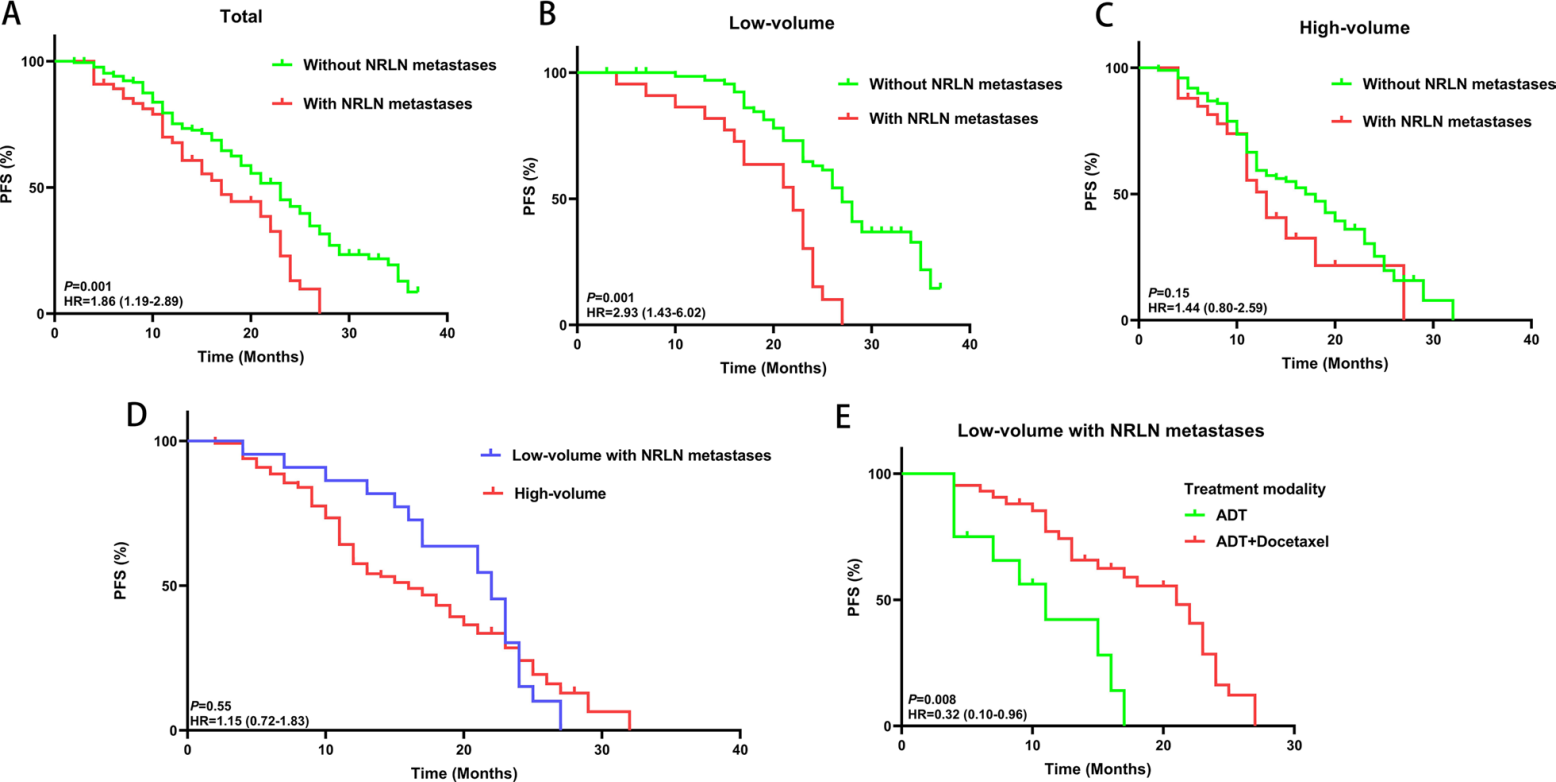
Kaplan‒Meier estimates of the impact of non-regional lymph node (NRLN) metastases on progression-free survival (PFS) for all patients with mHSPC in this study (**A**), patients with low-volume disease (**B**), and patients with high-volume disease (**C**). PFS was compared between patients with low-volume disease plus NRLN metastases and patients with high-volume disease (**D**). PFS was compared among patients with low-volume disease and NRLN metastases by different treatment arms (**E**)

Of the 224 patients, the median PFS of patients without NRLN metastases was 23.0 months, which was significantly longer than that of patients with NRLN metastases (23.0 vs. 17.0 months, *P* = 0.001) (Fig. 2A). Among patients with low-volume disease, the median PFS of patients with NRLN metastases was only 19.5 months, which was significantly shorter than that of patients without NRLN metastases (19.5 vs. 27.5 months, *P* = 0.001) (Fig. 2B). In the subgroup of patients with high-volume disease, the difference was not observed between patients with and without NRLN metastases (14.5 vs. 17.5 months, *P* = 0.15) (Fig. 2C). However, the difference in PFS between patients with low-volume plus NRLN metastases and high-volume disease was not significant (19.5 vs. 16.9 months, *P* = 0.55) (Fig. 2D). Furthermore, for patients with low-volume disease but concomitant NRLN metastases, ADT combined with early docetaxel chemotherapy significantly prolonged PFS compared with ADT alone (20.7 vs. 12.3 months, *P* = 0.008) (Fig. 2E).

## Discussion

Tumor volume is a critical factor for treatment decision-making in primary mHSPC. In addition to bone and visceral metastases, NRLN metastases may be an important additional factor for distinguishing between low- and high-volume diseases. In the current study, we focused on the cohort of primary mHSPC and found that the concordance rates of ^18^F-PSMA-1007 PET/CT and CI in detecting bone and visceral metastases were 82.83% and 86.87%, respectively, while the concordance rate in detecting NRLN metastases was only 61.62%. Among 94 patients without NRLN metastases on CI, NRLN metastases were detected by ^18^F-PSMA-1007 PET/CT in 37 patients (39.4%). Thus, we deduced that ^18^F-PSMA-1007 PET/CT had a higher sensitivity and specificity in detecting NRLN metastases than CI, which provided new insight into the clinical management of mHSPC.

Recently, PSMA PET/CT has shown increasing importance in the comprehensive management of PCa, including presurgical staging [22], biochemical recurrence prediction [23], and lymph node metastasis detection [24]. The diagnosis of lymph node metastases on CI mainly depends on the morphological size. However, the majority of metastatic nodes from PCa are < 8 mm [25], which does not satisfy the CI morphological size of lymph node metastases. Thus, tiny lymph node metastases of < 8 mm were inevitably missed by CI. Several studies have demonstrated that ^68^Ga-PSMA-11 PET/CT can provide a pattern of metastatic spread of lymph node metastases with higher sensitivity and specificity than CI and detect more NRLN metastases [26, 27]. Furthermore, a study conducted by Lenis et al*.* noted that the non-concordance rate between CI and ^68^Ga-PSMA-11 PET/CT was nearly 30%, and ^68^Ga-PSMA-11 PET/CT identified 20% of pelvic nodal disease and up to 10% of distant metastatic disease that was negative on CI [28]. To the best of our knowledge, this is the first study to evaluate the concordance rates between ^18^F-PSMA-1007 PET/CT and CI in terms of tumor volume evaluation and TNM classification. In the subset of patients who received ^18^F-PSMA-1007 PET/CT and CI before initial treatment, we found that ^18^F-PSMA-1007 PET/CT could detect more bone and visceral metastases that were negative on CI, but the effects of these additional findings on tumor volume evaluation were limited, as the concordance rate of tumor volume evaluation was 90.91%, and only 9 patients upgraded from low-volume to high-volume disease. Furthermore, Barbato et al. also noted that PSMA-PET criteria with volume quantification deliver comparable with CHAARTED criteria, with only 13% misalignment [29]. However, ^18^F-PSMA-1007 PET/CT detected an additional 37 of 94 (39.4%) patients with positive NRLNs who were negative on CI. Among 94 patients without NRLN metastases on CI, the median PFS of 37 patients with NRLN metastases on ^18^F-PSMA-1007 PET/CT was 18.0 months, which was significantly shorter than that of patients without NLRN metastases both on CI and ^18^F-PSMA-1007 PET/CT (18.0 vs. 25.0 months, *P* = 0.01) (Additional file 1: Fig. S1). In addition, 55 patients (58.5%) had diseases progression during the period of follow-up. Univariate Cox regression revealed that primary treatment modality, NRLN metastases detected by ^18^F-PSMA-1007 PET/CT were associated with PFS (Additional file 2: Table S1). Subsequently, the multivariate Cox analysis including all the above two variables suggested that ADT, NRLN metastases detected by ^18^F-PSMA-1007 PET/CT were also associated with worse PFS (all *P* < 0.05).

Furthermore, among 224 patients with primary mHSPC, we also found that NRLN metastases was an independent prognostic factor, and the progression risk of disease increased by twofold in patients with NLRN metastases. Ali et al*.* identified 17,167 patients who were diagnosed with mHSPC in the Surveillance, Epidemiology, and End Results database and noted that patients with bone and NRLN metastases had a significantly higher risk of all-cause mortality and prostate cancer-specific mortality [13]. Recently, Heesterman et al*.* also found that the presence of concomitant NRLNs and bone metastases was a poor prognostic sign, especially in patients with low-volume mHSPC [12]. Subsequently, a study also demonstrated that increased metastatic burden of 5 or more nodal metastases was associated with worse outcomes in patients with mHSPC either treated with ADT combined with docetaxel or ADT alone [30]. Moreover, our study further identified a special population with low-volume disease but with NRLN metastases. The PFS of these patients was 19.5 months, which was similar to that of high-volume disease but significantly shorter than that of patients with low-volume disease without NRLN metastases. This phenomenon warned that NRLN metastases, especially synchronous with bone metastases, should be considered a high-risk feature with metastatic volume stratification definitions to improve the accuracy of disease volume-based treatment decisions.

The management of NRLN metastases remains a challenge for urologists, and metastatic volume evaluation might be helpful. Tumor volume classification, as proposed in the CHAARTED trials, is universally accepted for use in clinical practice or clinical trial conduct. For primary mHSPC synchronous with NRLN metastases, several studies have shown that local treatment combined with systemic therapy could improve the prognosis compared with systemic therapy alone [31]. However, we should pay more attention to interpreting these results, as the potential benefit of local treatment depends largely on the characteristics of the primary tumor and the number of suspicious nodes [32]. Prospective data from arm H of the STAMPEDE trial showed that the survival benefit of prostate radiotherapy decreased continuously as the number of bone metastases increased, with the benefit most pronounced with up to three bone metastases [33]. Furthermore, they also noted that the magnitude of benefit from the addition of prostate radiation therapy was greater in patients with only NRLN metastases, which indicated that only patients with very low metastatic burden (only NRLN metastases) or oligometastatic status (three or fewer bone metastases without visceral metastasis) might benefit more from local treatment. Previously, the CHAARTED trial showed that docetaxel could improve the prognosis for mHSPC patients with high-volume disease, but the benefit of docetaxel was not observed for patients with low-volume disease [7]. As mentioned above, coexisting NRLNs and bone metastases represented a high metastatic burden, even in patients with low-volume disease. Recently, the 2023 EAU guidelines recommended that all men with mHSPC should receive intensified ADT + androgen receptor signaling inhibitors (ARSI) or triple therapy (ADT + ARSI + docetaxel) [15]. However, the most suitable population for triple therapy is still unknown, and multi-drug combination therapy might result in more toxicity events. Thus, ADT combined with docetaxel or ARSI was still first-line treatment option for patients with primary mHSPC in clinical practice. Previously, several studies demonstrated that ADT combined docetaxel could improve the prognosis for mHSPC patients with high-volume disease, but the benefit of docetaxel was not observed for patients with low-volume disease. In the present study, we found that patients with low-volume plus NRLN metastases had a poor prognosis and could benefit more from docetaxel than ADT alone. Herein, we suggested that the metastatic burden of patients with low-volume plus NRLN metastases was remarkably higher than that of patients with low-volume without NRLN metastases but similar to that of patients with high-volume disease, and early intensive treatments, such as docetaxel, should be performed for these patients.

Despite these positive results in this real-world retrospective study, there also had several limitations. First, the retrospective nature of the present study and the small sample size from a single center might cause selection bias. Second, a part of patients only received CI examination, might indicate insufficient metastatic screening and tumor volume evaluation. Moreover, due to the short follow-up time, the impact of NRLN metastases on overall survival was not analyzed, and a longer follow-up duration might produce more positive findings. Lastly, only less of half the patients in our study had both received ^18^F-PSMA-1007 PET/CT and CI before treatment and there was no standard of reference for the assessment of diagnostic performance in our study. Hence, further prospective multicenter studies are needed to support our findings.

## Conclusions

In the present study, we found that the concordance rates between ^18^F-PSMA-1007 PET/CT and CI in terms of tumor volume evaluation were comparable. However, the concordance rate between ^18^F-PSMA-1007 PET/CT and CI in detecting NRLN metastases was very low, as ^18^F-PSMA-1007 PET/CT could reveal more NRLN metastases, which was an important prognostic factor for primary mHSPC. Furthermore, in patients with low-volume disease, the presence of concomitant NRLN metastases is a poor prognostic sign compared to patients without NRLN metastases, and more intensive treatment, such as early docetaxel chemotherapy, may be more suitable for these patients.

## Supplementary Information

**Additional file 2.** **Fig. S1.** The progression-free survival (PFS) difference between patients with and without non-regional lymph node (NRLN) metastases on 18F-PSMA-1007 PET/CT in patients with negative NRLN metastases on CI.

**Additional file 2.** **Table S1.** Cox regression analysis for associations of clinicopathological parameters with PFS among 94 patients without NRLN metastases on CI.

## Data Availability

The datasets generated during the current study are available from the corresponding author on reasonable request.

## Abbreviations

NRLN: Non-regional lymph node
mHSPC: Metastatic hormone-sensitive prostate cancer
CI: Conventional imaging
PFS: Progression-free survival
ADT: Androgen deprivation therapy
PCa: Prostate cancer
CT: Computerized tomography
MRI: Magnetic resonance imaging
PSMA: Prostate-specific membrane antigen
GS: Gleason score
PSA: Prostate-specific antigen
