# Supplementary figures and images for: Impact of non-regional lymph node metastases accurately revealed on 18F-PSMA-1007 PET/CT in the clinical management of metastatic hormone-sensitive prostate cancer
Source: EJNMMI Res. 2023 Jul 6;13:64. doi: 10.1186/s13550-023-01009-x (PMC10326178; doi:10.1186/s13550-023-01009-x)

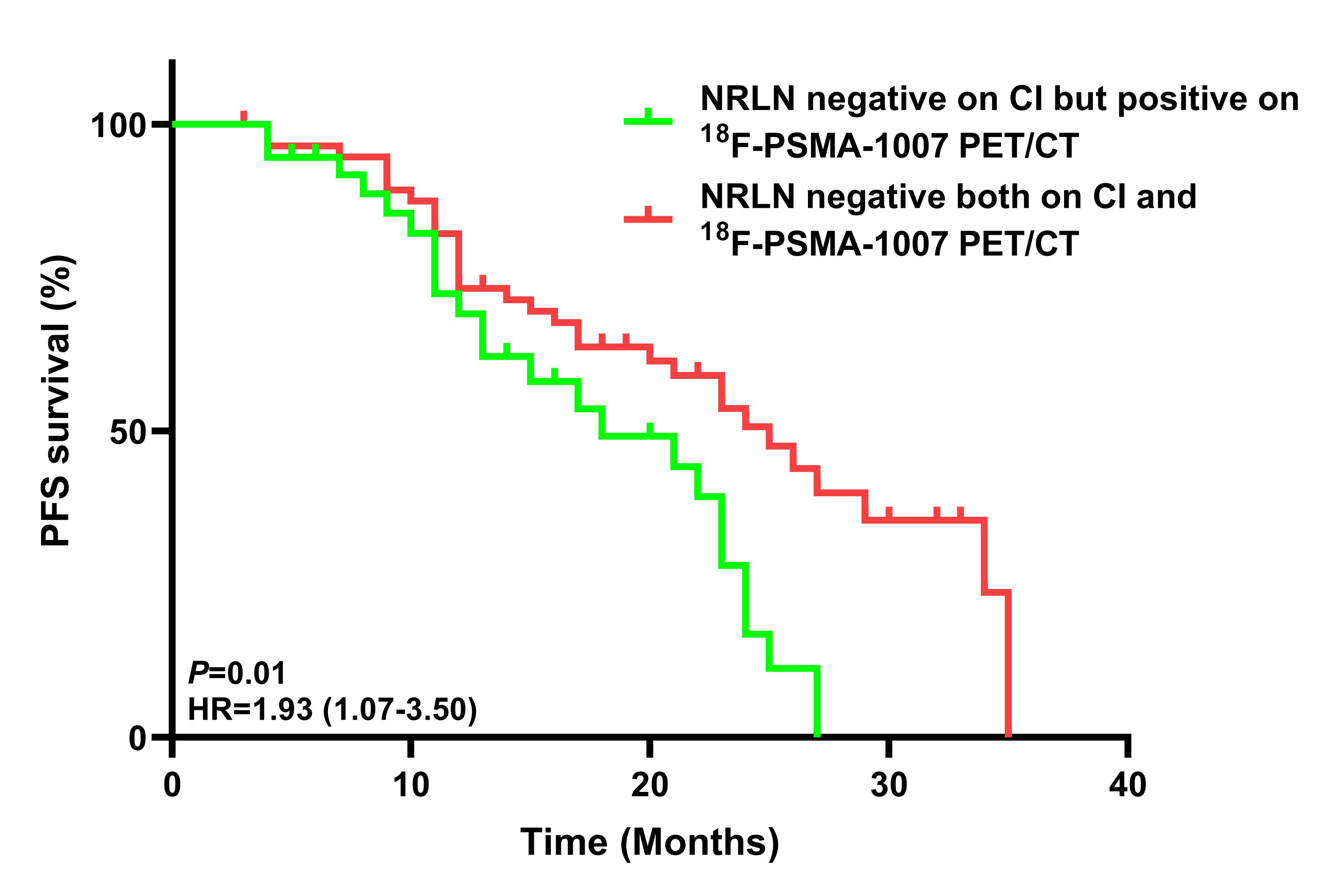

Supplement: Supplementary file 1 — Additional file 2. Fig. S1. The progression-free survival (PFS) difference between patients with and without non-regional lymph node (NRLN) metastases on 18F-PSMA-1007 PET/CT in patients with negative NRLN metastases on CI. [file 13550_2023_1009_MOESM1_ESM.jpg]
