# Supplementary material for: Impact of non-regional lymph node metastases accurately revealed on 18F-PSMA-1007 PET/CT in the clinical management of metastatic hormone-sensitive prostate cancer
Source: EJNMMI Res. 2023 Jul 6;13:64. doi: 10.1186/s13550-023-01009-x (PMC10326178; doi:10.1186/s13550-023-01009-x)
Supplement: Supplementary file 2 — Additional file 2. Table S1. Cox regression analysis for associations of clinicopathological parameters with PFS among 94 patients without NRLN metastases on CI. [file 13550_2023_1009_MOESM2_ESM.docx]

**Additional file 2**

**Table** S1 Cox regression analysis for associations of clinicopathological parameters with PFS^e^ among 94 patients without NRLN^c^ metastases on CI^e^.

| Variable | Univariable | | |  | Multivariable | | |
| --- | --- | --- | --- | --- | --- | --- | --- |
|  | HR | 95%CI | *P* |  | HR | 95%CI | *P* |
| Age (Continuous) | 0.997 | 0.962-1.034 | 0.890 |  |  |  |  |
| PSA^a^ at diagnosis (Continuous) | 1.034 | 0.764-1.523 | 0.261 |  |  |  |  |
| Biopsy Gleason score |  |  |  |  |  |  |  |
| 8 vs.6-7 | 1.106 | 0.524-2.334 | 0.792 |  |  |  |  |
| 9-10 vs. 6-7 | 1.004 | 0.533-1.890 | 0.990 |  |  |  |  |
| Primary treatment modality (ADT^b^ vs. ADT+Docetaxel) | 0.449 | 0.245-0.823 | 0.010 |  | 0.317 | 0.165-0.611 | 0.001 |
| Tumor volume (Low vs. High) | 0.689 | 0.393-1.209 | 0.194 |  |  |  |  |
| T stage |  |  |  |  |  |  |  |
| cT3 vs. cT2 | 1.066 | 0.514-2.211 | 0.864 |  |  |  |  |
| cT4 vs. cT2 | 1.035 | 0.564-1.899 | 0.913 |  |  |  |  |
| N stage (N0 vs. N1) | 0.561 | 0.209-1.067 | 0.084 |  |  |  |  |
| NRLN^c^ metastatses (absence on ^18^F-PSMA^d^-1007 PET/CT vs. presence on ^18^F-PSMA-1007 PET/CT) | 0.491 | 0.279-0.862 | 0.013 |  | 0.363 | 0.198-0.667 | 0.001 |
| Bone metastases (absence vs. presence) | 0.722 | 0.307-1.701 | 0.456 |  |  |  |  |
| Visceral metastases (absence vs. presence) | 0.805 | 0.250-2.591 | 0.716 |  |  |  |  |

1. PSA: prostate specific antigen; b. ADT: androgen deprivation therapy; c. NRLN: non-regional lymph node; d. PSMA: prostate specific membrane antigen; e. CI: conventional imaging;
